# Accessing Patient Electronic Health Record Portals Safely Using Social Credentials: Demonstration Pilot Study

**DOI:** 10.2196/29647

**Published:** 2022-01-27

**Authors:** Spencer SooHoo, Michelle S Keller, Harold Moyse, Benjamin Robbins, Matthew McLaughlin, Ajay Arora, Abigail Burger, Lilith Huang, Shao-Chi Huang, Anil Goud, Lyna Truong, Donaldo Rodriguez, Pamela Roberts

**Affiliations:** 1 Enterprise Information Systems Cedars-Sinai Medical Center Los Angeles, CA United States; 2 Division of Informatics Department of Biomedical Sciences Cedars-Sinai Medical Center Los Angeles, CA United States; 3 Division of General Medicine Depart of Medicine Cedars-Sinai Medical Center Los Angeles, CA United States; 4 Select Medical Mechanicsburg, PA United States; 5 California Rehabilitation Institute Los Angeles, CA United States; 6 Department of Physical Medicine and Rehabilitation Cedars-Sinai Medical Center Los Angeles, CA United States

**Keywords:** patient portal access, single sign-on, federated identity, social credentials, social identity, patient portal, electronic health records, EHR, credentials, patient communication, communication, clinical support, feasibility, acceptability

## Abstract

**Background:**

Patient portals allow communication with clinicians, access to test results, appointments, etc, and generally requires another set of log-ins and passwords, which can become cumbersome, as patients often have records at multiple institutions. Social credentials (eg, Google and Facebook) are increasingly used as a federated identity to allow access and reduce the password burden. Single Federated Identity Log-in for Electronic health records (Single-FILE) is a real-world test of the feasibility and acceptability of federated social credentials for patients to access their electronic health records (EHRs) at multiple organizations with a single sign-on (SSO).

**Objective:**

This study aims to deploy a federated identity system for health care in a real-world environment so patients can safely use a social identity to access their EHR data at multiple organizations. This will help identify barriers and inform guidance for the deployment of such systems.

**Methods:**

Single-FILE allowed patients to pick a social identity (such as Google or Facebook) as a federated identity for multisite EHR patient portal access with an SSO. Binding the identity to the patient’s EHR records was performed by confirming that the patient had a valid portal log-in and sending a one-time passcode to a telephone (SMS text message or voice) number retrieved from the EHR. This reduced the risk of stolen EHR portal credentials. For a real-world test, we recruited 8 patients and (or) their caregivers who had EHR data at 2 independent health care facilities, enrolled them into Single-FILE, and allowed them to use their social identity credentials to access their patient records. We used a short qualitative interview to assess their interest and use of a federated identity for SSO. Single-FILE was implemented as a web-based patient portal, although the concept can be readily implemented on a variety of mobile platforms.

**Results:**

We interviewed the patients and their caregivers to assess their comfort levels with using a social identity for access. Patients noted that they appreciated only having to remember 1 log-in as part of Single-FILE and being able to sign up through Facebook.

**Conclusions:**

Our results indicate that from a technical perspective, a social identity can be used as a federated identity that is bound to a patient’s EHR data. The one-time passcode sent to the patient’s EHR phone number provided assurance that the binding is valid. The patients indicated that they were comfortable with using their social credentials instead of having to remember the log-in credentials for their EHR portal. Our experience will help inform the implementation of federated identity systems in health care in the United States.

## Introduction

### Background

Providers and patients operate within a complex and fragmented health care environment. Challenges in delivering and receiving care across distinct health care organizations (eg, primary care clinics, specialty clinics, hospitals, and psychiatric facilities) require the exchange of information and access to organizationally distinct information systems. Electronic health record (EHR) software is being increasingly adopted by hospitals and health care entities. The promotion of provider and patient involvement in the delivery of health care for quality and safety of care is of critical importance [1,2]. Furthermore, government incentive programs and regulations have influenced health care organizations to implement patient access [3]. With the increasing need for access to the EHR, user account credential management has become a growing problem. A Microsoft study found that an average user has 25 different accounts and uses 6.6 passwords shared across 3.9 sites [4]. The study also found that a user types an average of 8 passwords per day. Furthermore, a McAfee survey reported that the average consumer deals with 23 web-based accounts that require a password [5]. The same survey showed that each user had an average of 13 unique passwords, and 31% used only 2 to 3 passwords for their accounts so that they could easily remember them. Approximately 52% reported that writing down passwords (either on paper or digitally) is the most common way to remember them.

Forgotten passwords are problematic, as illustrated in a press report referencing a joint Mastercard and Oxford University study [6]. The study found that 25% of consumers had 1 password reset per day and that 33% of shopping carts for web-based purchases were abandoned at checkout because of password problems. In addition, another study reported that 78% of respondents required a password reset in their personal life in the past 90 days, and 57% required a work password reset in the past 90 days [7]. The same study found that over one-third of the respondents had >20 passwords for their personal life, and almost 20% had >10 work-related passwords.

Another factor that adds to the friction associated with access is that some sites require strong passwords (typically a minimum of 8 characters with numbers, upper or lower cases, and often a special character) and may also require periodic password changes, despite a recent National Institutes of Standards and Technology (NIST) recommendation against complexity and password expiry in favor of long passphrases [8]. The NIST recommends the use of multifactor authentication (MFA) in conjunction with a passphrase that does not expire unless there is reason to believe that the password or passphrase has been compromised [8].

The number of EHR patient portals is rising in the United States largely because of the US Electronic Health Record Incentive Program and Meaningful Use [9] and patients’ desires to make appointments on the web, communicate with their providers, request appointments and medication refills, and view test results [10-12]. For example, a 2019 study exploring hematology patients’ desires for a patient portal found that a large proportion of patients (>75%) wanted the ability to contact their physicians and access laboratory tests, imaging results, appointments, personal data, current medication lists, medication history, and reports to other physicians [10]. A large proportion of patients (>75%) also wanted the ability to make appointments, set up appointment reminders, request medication refills, change their personal data, and access medication and disease information [10]. During the COVID-19 pandemic, patient portals have served as a way for patients to self-triage and self-schedule appointments based on their needed level of care [13] and access vaccines [14].

In the United States and in countries with similarly fragmented health systems, for an individual or their family, having their health care records spread out among multiple facilities causes fragmentation [15,16] and does not provide the health care provider or individual with comprehensive information in an EHR. Patients are often faced with having multiple log-in credentials for each facility where they had received health care services.

Although access to and aggregation of patient data from multiple sources is rapidly evolving, there are 3 general approaches [17]. There are directed exchanges where health provider organizations (HPOs) send and receive information to coordinate care, query-based exchanges where one HPO queries another for information on a specific patient, and consumer-mediated exchanges where patients can direct and control the aggregation and use of their information. Proprietary data exchanges, typically within an EHR vendor’s technology ecosystem, allow data to be exchanged among HPOs who use the same EHR vendor. Examples are Epic’s Care Everywhere *Happy Together* [18] and Cerner’s *HealtheLife* [19]. These are patient portals that allow patients to see all their EHR data across different HPOs that use the same EHR vendor with a single log-in. However, if, for example, a patient had EHR data in an Epic system and another set of data in a Cerner system, the patient would have to use 2 different EHR patient portals, each with their own log-in credentials and password complexity requirements. This makes it cumbersome to easily obtain one’s health information and requires remembering multiple passwords. A federated credential would allow patients to get the source data with a single log-in.

Health information exchanges allow providers to share health information, although they may use different EHR systems, and other data-sharing initiatives such as CommonWell Health Alliance [20] have the potential to allow patients to self-enroll from a patient portal or personal health record (PHR) and access all of their EHR data across multiple HPOs regardless of the EHR system used. Again, this somewhat reduces the burden of additional credential management; however, it can be further reduced using a federated identity that the patient is comfortable with and uses frequently.

Substitutable Medical Applications, Reusable Technologies (SMART) on Fast Healthcare Interoperability Resource (FHIR) apps [21] allow patient-directed EHR data sharing. After successful authentication at each portal, SMART on FHIR apps access EHR data from each participating HPO and allow them to be downloaded or shared with a third party. Two examples are the Apple Health app [22], which is representative of a SMART on FHIR solution that allows patients to download EHR data to a mobile device, and the Sync for Science app [23], a pilot to demonstrate the ability to share a patient’s EHR data for the direct volunteer cohort of the National Institutes of Health–sponsored All of Us Research Program [24]. Again, although these solutions have the potential to reduce patients’ password burden, they may increase the burden if they require their own unique credentials, and patients may still have to contend with different log-in credentials for each EHR portal when first setting up the SMART on FHIR app unless they support federated log-in credentials. In an ideal scenario, the same federated credentials could be used for the EHR portal and to access the SMART on FHIR app.

A common way to reduce the friction associated with passwords on mobile devices is the use of biometric attributes such as fingerprint or facial recognition for access. On the surface, these approaches make access simpler; however, an underlying problem is that log-in credentials are required at the initial configuration, and a software upgrade or device reset may erase the cached password and prompt the user to enter credentials that likely have been forgotten since, especially if it is one of many that are infrequently used.

**Figure 1:**
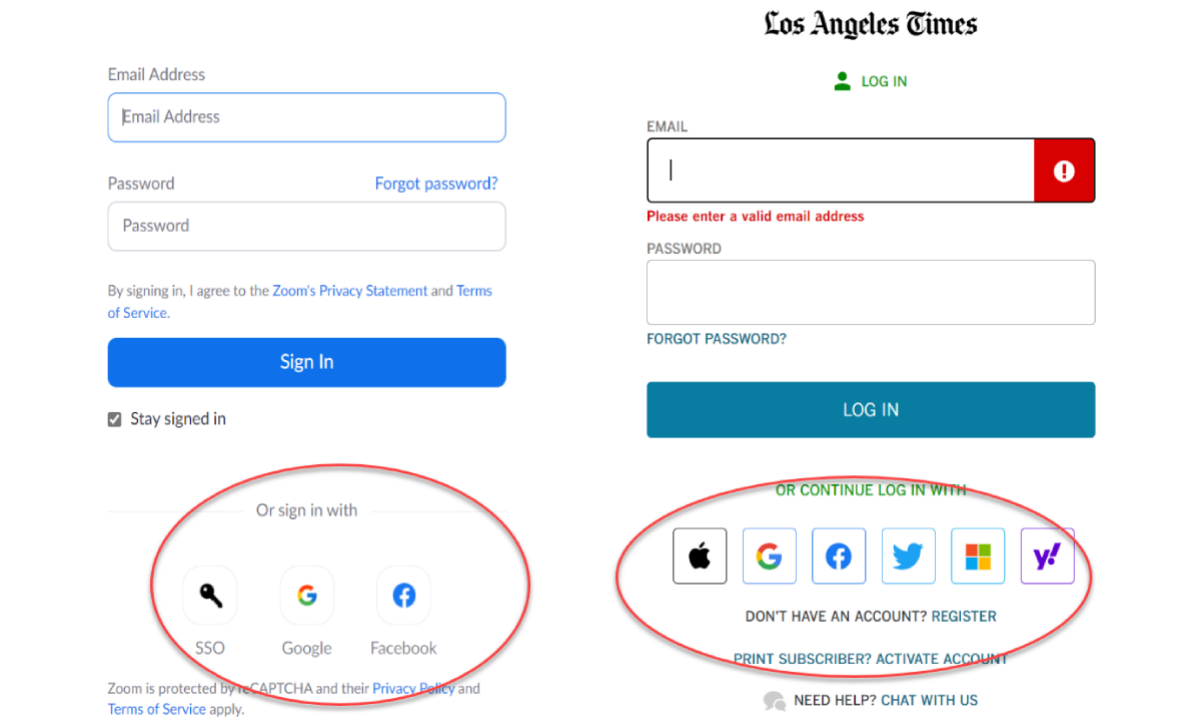
Two samples of single sign-on using a social credential. The Zoom login on the left allows a Google or Facebook credential, while subscribers to the Los Angeles Times (right) can use an Apple, Google, Facebook, Twitter, Microsoft, or Yahoo account for single sign-on.

A single sign-on (SSO) approach that relies on a secure federated identity and associated credentials can reduce the friction associated with EHR data access [8,25,26]. Such approaches are increasingly common in the non–health care arena, as illustrated in Figure 1, which shows the use of social credentials such as Facebook, Google, or Twitter to access a newspaper portal and videoconferencing. As these examples illustrate, SSO allows a user to use a single credential to have access to resources among different organizations. SSO used with a federated identity is predicated on having multiple distinct organizations that agree to a common set of practices, policies, and protocols to manage a single identity. This identity is trusted to access services and devices across participating organizations [27]. In a 2017 report, NIST noted that federated architectures have significant benefits, including enhanced user experience, cost reductions because of fewer authenticators needed, data minimization, and mission enablement, as organizations can focus on their mission as opposed to the business of identity management [8].

As web-based SSOs are becoming a common technique that allows users to easily self-register and sign onto web-based resources using social media accounts, NIST and the Office of the National Coordinator for Health Technology (ONC) selected the Cedar-Sinai Medical Center (CSMC) to pilot the use of a federated identity to access EHR data across multiple independent HPOs for both patients and providers and assist with the development of a *lessons learned* document. The primary requirements for the pilot were as follows:

- Implement SSO for EHR access at ≥2 distinct health systems using a federated, verified identity based on effective identity-proofing processes
- Allow use of pseudonymous identities
- Use MFA
- Incorporate privacy-enhancing technology
- Collaborate with NIST and ONC representatives to develop a lessons learned document that can inform future deployments of federated identity solutions in health care in the United States

In addition to the strict software development efforts required to implement a federated identity management solution, other types of barriers include the following:

1. Technology standards: This covers interoperability among the infrastructure components of each federation partner and the choice of standards (such as OpenID Connect, OAuth 2, and security assertion markup language) and vendor-specific implementation of standards.
2. Governance: This requires acceptance of a trust framework whereby the members of a federation agree to their respective roles and responsibilities, determine what type of information can be exchanged, what safeguards are needed, and dispute resolution procedures.
3. Legal: There are state and federal laws specific to the exchange of health records, including the requirements for security and privacy controls. For this pilot, our software had to accommodate the use of proxies (often caregivers or family members) for patients, and we conducted several security and privacy reviews to minimize risks to patient identifiers.
4. Organizational constraints: These include organizational priorities, staffing, and budgets that affected the deployment of the pilot.

### Objective

The objective of this project is to demonstrate, in a real-world environment, that it is possible to overcome both software and nonsoftware barriers to the adoption of a federated identity for patient EHR access and to enroll actual patients to test the concept.

The use case selected for a real-world test of this pilot project was inpatient transitions from a US-based acute care hospital to a US-based inpatient rehabilitation facility. This has been a focus of attention [28] because of its inherent vulnerable population—individuals with high levels of care needed after discharge from a hospital—that are at risk for morbidity and mortality, resulting too often in readmissions. The lack of information access is complicated by the fact that many patients and providers must access ≥2 distinct EHR systems for information retrieval and continuity of care.

Therefore, the primary aim of this study is to implement a Single Federated Identity Log-in for EHRs (Single-FILE) to facilitate access to EHR data on distinct systems at multiple health care institutions for patients via federated identities and SSO, with both EHR portals visible at the same time. Many EHR implementations rely primarily on passwords as a primary security control; however, these credentials may have been unknowingly stolen. Single-FILE incorporates 2 features to minimize this risk when patients access EHR data via Single-FILE. When a patient is on-boarded into Single-FILE, a one-time passcode (OTP) is sent to the phone number previously registered in the patient’s EHR record to confirm the legitimacy of the username and password log-in.

In this paper, we describe the technological approach we used to develop and implement the Single-FILE web portal. We then present the methods and results of interviews with patients who signed up for and used the webportal and provided feedback. We conclude with lessons learned from this pilot, which have broad applicability beyond the current project.

## Methods

### Security and Privacy

One of the sponsors’ primary requirements was that any architecture we arrived at had to be privacy-preserving, so our approach was to avoid storing any direct patient identifiers, demographics, or other EHR data in Single-FILE. Information security was also an important consideration, so the project team conducted multiple design workshops that were attended by an external information security consultant and the CSMC chief privacy officer. These were supplemented by design review workshops with the NIST and ONC staff. When the development was finished, automated security scans were performed on the Single-FILE components, and these were augmented by 2 independent, manual penetration tests, and vulnerabilities were corrected.

A supplement to the security scans and penetration tests was the requirement by NIST that we use a privacy risk assessment methodology (PRAM) [29] and that the results and remediation be reviewed and approved by our chief privacy officer. The PRAM is a holistic systematic review of a system that requires an analysis of the path that each data element takes in the system and an assessment to determine the possibility of the breach of the data element and the harm that could result from the breach. For data elements where the risk of breach and harm is great, mitigating controls must be implemented before the go-live. We worked with the CSMC chief privacy officer, who indicated that such a structured approach was relatively novel and that much of her focus (and of her peers’ focus) is still on compliance with regulations rather than taking a holistic, structured analysis of a system to uncover potential privacy risks.

### Technological Approach

Although we provided a way for patients to self-register an account managed by Single-FILE, we also allowed the patient to select a social identity to use as a federated identity. In either case, we bound the identity to the corresponding EHR identity.

**Figure 2:**
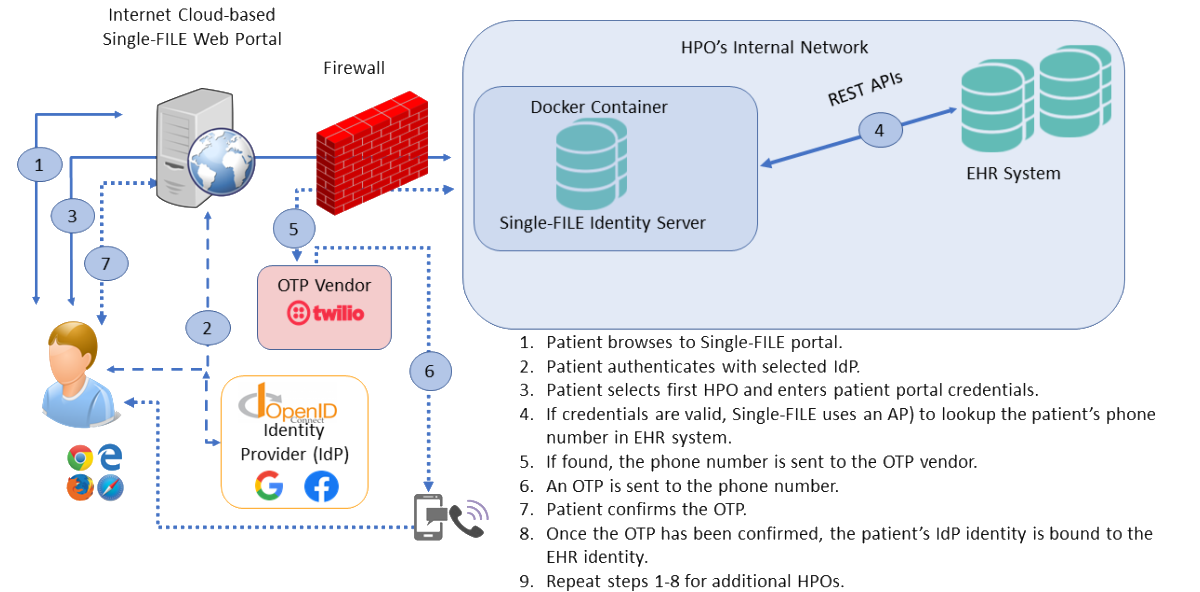
Single Federated Identity Log-in for electronic health records architectural overview. API: application programming interface; HPO: Health care provider organization; IdP: identity provider; OTP: one-time passcode; REST: Representational State Transfer; Single-FILE: Single Federated Identity Log-in for electronic health records.

Binding a social identity requires that the social identity be legitimate, and, following the OAuth 2 protocol [30], the patient must successfully enter their social identity credentials. For this project, Facebook and Google were selected as the social identity providers (IdPs). As illustrated in Figure 2, the patient accesses the Single-FILE webportal and, upon selecting an IdP, is redirected to the selected IdP’s log-in screen. Upon a successful log-in, the IdP returns an authorization token as evidence that the social identity is valid.

The next step is to bind the social identity to the patient’s EHR record. Patients were prompted to enter their EHR portal credentials at the Single-FILE portal, and upon successful log-in to the EHR patient portal, the patient’s phone number that was previously recorded in the EHR (typically when the patient was admitted and an EHR record created) was retrieved. As there is a risk that a malicious actor could have knowledge of the patient’s social and EHR credentials, an OTP is sent to the phone number stored in the EHR (either voice or SMS text message, depending on the type of phone). Once the patient acknowledges the OTP, the social identity is bound to the EHR identity. Essentially, the phone is used as a *token* that the patient has control of and is used as an additional authentication factor.

The Single-FILE portal was installed on an Amazon Web Services instance, and the identity server was installed at each HPO as a Docker container [31], which is a way of packaging up software along with all the environmental dependencies (code, system tools, system libraries, and settings) so that everything is contained in a ready-to-execute software package. This ensures that the software will run despite the differences between the development and development settings. The Docker container (1) is deployed inside each HPO’s network perimeter and provides the connection between the Single-FILE webportal and each respective HPO’s EHR system, (2) preserves privacy by containing only the minimum necessary patient information to operate, and (3) provides a way to ensure that the Single-FILE software is not changed by a malicious actor.

Single-FILE was deployed and tested with patients who were discharged from CSMC and admitted to the California Rehabilitation Institute (Cal Rehab). Both CSMC and Cal Rehab use Epic for their EHR vendors; however, the concept can be readily extended to other EHR vendors.

### Participants and Setting

Our study involved patients who received care at an acute care hospital in Los Angeles, California (CSMC), who were discharged and immediately admitted to an inpatient rehabilitation hospital (Cal Rehab), also located in Los Angeles, California. The acute care hospital, CSMC, serves the Los Angeles community with 886 licensed beds, 2100 physicians in every clinical specialty, 2800 nurses, and thousands of other health care professionals, staff, and volunteers. The CSMC has approximately 90,000 emergency department visits, 50,000 admissions, and 17,000 inpatient and 13,000 outpatient surgeries per year. The health system is an academic medical center with trainees in medicine, nursing, pharmacy, public health, and clinical and basic science. Cal Rehab is a 138-bed inpatient physical medicine and rehabilitation hospital located in Los Angeles. It is a partnership between CSMC, University of California Los Angeles Health System, and Select Medical. Patients at Cal Rehab have intensive rehabilitation needs for conditions such as spinal cord injury, brain injury, orthopedic surgery, and stroke and work with physical medicine and rehabilitation physicians as well as physical and occupational, and speech-language pathologists.

### Real-world Evaluation of Federated Identity for EHR Access by Patients

#### Patient Recruitment and Interviews

To identify potentially eligible patients to test Single-FILE, we identified patients recently admitted to the rehabilitation hospital (Cal Rehab) who consented to research. Patients are asked on admission to Cal Rehab if they would be interested in consenting to research, and a list is generated daily of patients who have consented to research. This list and a list of patients recently admitted to the facility were obtained by one of the investigators (PR) who oversaw research initiatives at the rehabilitation hospital. Before approaching patients, we conducted a short chart review to examine whether they previously had an inpatient stay at the acute care hospital (CSMC) preceding their stay at Cal Rehab and whether they had not yet been discharged from Cal Rehab, as some rehabilitation stays are short. Initially, we aimed to enroll patients with existing patient portals; however, as the patient population of inpatient rehabilitation hospitals skews toward older adults, and this population is less likely to have a patient portal [32], we expanded our eligibility criteria to patients who stayed at both locations but did not require that the patient had a patient portal. A qualitative researcher (MSK) approached patients and caregivers (if the patient was unable to communicate) at Cal Rehab and discussed the study objectives and procedures. If patients and caregivers (or both) consented to participate, we worked with patients and caregivers to set up a patient portal log-in at CSMC and Cal Rehab (if needed) and registered them in the Single-FILE portal. We subsequently conducted a short 15- to 20-minute in-person interview using a semistructured interview guide, which included a short demographic questionnaire. Topics in the guide included a short description of the patients’ condition and experiences in the acute and postdischarge settings, experiences transitioning from one facility to another, previous use of a patient portal, preferred functionality in patient portals, and use of the internet to access health information. The demographic questionnaire included questions about gender, race, ethnicity, education level, marital status, health status, level of interest in using the internet to manage health care (high, some, none, do not know or need more information) and health literacy (question: “How confident are you in filling out medical forms on your own?” answers: “Not at all, a little bit, somewhat, quite a bit, extremely”). We also called patients 30 days after their enrollment in the study to explore their use of Single-FILE.

#### Qualitative Analysis

Framework analysis and open coding were used to analyze qualitative data. This methodology includes the transcription of the data, thorough reading of each transcript, coding of the data using open coding development of an analytical framework, applying the analytical framework or codebook, charting the data using a framework matrix, and interpreting the data.

## Results

### Implementation Barriers

As part of the implementation of Single-FILE for a real-world pilot, there was a need for cooperation and coordination with our implementation partner, Cal Rehab. These are documented in a *lessons learned* document published by the ONC and fall into 4 broad categories.

#### Technology Standards

This specific pilot was limited to 2 sites using independent Epic implementations. We encountered delays because the application programming interfaces (APIs) used by Single-FILE were dependent on features of a version of Epic that was not implemented in earlier versions. At the start of the project, we anticipated that Cal Rehab would upgrade to the current version of Epic; however, they made a business decision to skip to the next upgrade, delaying the project by over 12 months.

In addition, in our case, both CSMC and Cal Rehab used Epic as the EHR vendor. If Cal Rehab was using another vendor, the web service calls would have to be modified. While this pilot was underway, integration based on FHIR advanced rapidly, and as discussed later, the use of FHIR calls for patient identity verification, and binding eliminates EHR vendor-specific software and provides a standards-based API.

#### Governance

For any federated identity to be acceptable, the participating parties must be able to trust that other parties adhere to the same security and privacy standards. A trust framework document spells out the responsibilities of each party. Our work revealed that for our (CSMC) environment, the idea of a trust framework was novel to our health system leaders, and our chief privacy officer indicated that this was likely true for other health systems.

#### Legal

EHR access by proxies was a requirement for patients who were unable to access their EHR data on their own. Although this was not a major challenge to implement, we also had to ensure that we were compliant with several federal and state regulations to help ensure the privacy and confidentiality of patient information. The security scans, penetration tests, and PRAM review provided a high level of assurance that we would meet state and federal privacy and confidentiality standards.

#### Operations

The expertise and authority needed to make decisions are compartmentalized within organizations and vary among organizations, and the implementation staff are not necessarily aware of or able to influence policy decisions. In addition, implementation targets and timelines were heavily affected by organization priorities. As mentioned earlier, the Single-FILE platform architecture relied on specific Epic web service calls; however, Cal Rehab was 1 version behind and did not support the needed web service calls. Cal Rehab made the decision to skip the upgrade and wait until the next version, delaying implementation by approximately 1 year.

Other delays were encountered as security and interface configurations were controlled by different, siloed teams within the same organization; thus, there were delays in making configuration changes and troubleshooting sessions to identify problems. An additional complication was the outsourcing of parts of the EHR infrastructure, which further impeded troubleshooting and tuning the final configuration.

### SSO Acceptability

A total of 8 patients and their caregivers were interviewed at Cal Rehab. Most patients did not have an existing portal log-in for at least one site. Sign-up for the patient portal or portals and Single-FILE took approximately 60 to 90 minutes. This included the time needed to explain the project and get signed consent. The demographic characteristics of the patients are presented in Table 1. The patients enrolled were predominantly White (7/8, 88%) and non-Hispanic (5/8, 62%). Half of the patients or their caregivers reported that the patient had *fair* health. Most patients and their caregivers reported *high* or *some* level of interest in using the internet to manage their health. Health literacy levels were distributed throughout the spectrum, with 25% (2/8) of patients and their caregivers noting low levels of health literacy, 38% (3/8) reporting some levels of health literacy, and 25% (2/8) reporting *quite a bit* or *extremely* high levels of health literacy.

Approximately three-fourths of the patients and caregivers reported already using the Epic patient portal (MyChart). The most common uses of the patient portal included being able to track or change upcoming appointments, reviewing laboratory or test results, or contacting the clinician directly. A patient noted that he used the portal to look at the visit notes and explained the following:

Oh, I like being able to just pop on and schedule an appointment, and check appointments, change them. See any of my tests that have been run for me, or referrals, and really all of it. I don’t want paper, and I don’t want to make a phone call, if I can save it.

Patient 1

Another patient noted that he liked to have access to his medical information quickly:

I knew from my doctor’s office that they had a portal that I could sign up with that I could add, all my doctors would be added to it. All my appointments would be added to it. All my MRIs, CAT scans, lab work, the reports would all go on to that so I could look at it before my doctor even called me. And I like to have information as quick as possible.

Patient 2

Of those who did not regularly use the patient portal before signing up for Single-FILE, barriers included not feeling comfortable navigating the internet or using technology overall. One patient who did not use the patient portal noted the following:

I hardly use the internet. I really don’t.

Patient 3

This patient relied on her caregiver, a sibling, to access the patient’s portal.

Patients noted that they appreciated only having to remember 1 log-in as part of Single-FILE and being able to sign up through Facebook. However, we did not see the use of Single-FILE by patients after they signed up. We attempted to reach patients and their caregivers via phone calls 30 days post sign-up but were not able to interview individuals, as this time coincided with the beginning of the COVID-19 pandemic, and the individuals reached did not want to participate in interviews at that time.

**Table 1:** Patient demographics and characteristics (N=8).

Characteristics			Values
Age (years), mean (SD)			65 (16.3)
**Gender, n (%)**			
	Male	5 (62)	
	Female	3 (38)	
**Race, n (%)**			
	White	7 (88)	
	Black	1 (12)	
	Other	0 (0)	
**Ethnicity, n (%)**			
	Hispanic	1 (12)	
	Non-Hispanic	5 (62)	
	Other	2 (25)	
**Education, n (%)**			
	Less than high school, high school, or General Educational Development	0 (0)	
	Some college	4 (50)	
	College	3 (38)	
	Graduate school	1 (12)	
**Marital status, n (%)**			
	Single	2 (25)	
	Married	4 (50)	
	Widowed	1 (12)	
	Divorced	1 (12)	
	Domestic partnership or cohabiting with partner	0 (0)	
**Self-reported health, n (%)**			
	Excellent	0 (0)	
	Very good	2 (25)	
	Good	2 (25)	
	Fair	4 (50)	
	Poor	0 (0)	
**Internet health literacy (what is your level of interest in using the internet to manage your health?), n (%)**			
	High	3 (38)	
	Some	4 (50)	
	None	1 (12)	
	Do not know or need more information	0 (0)	
**Health literacy (how confident are you in filling out medical forms on your own?), n (%)**			
	Not at all	2 (25)	
	A little bit	0 (0)	
	Somewhat	3 (38)	
	Quite a bit	1 (12)	
	Extremely	1 (12)	

## Discussion

### Principal Findings

The implementation of Single-FILE demonstrated that it is possible to safely bind a social identity to an EHR identity. The use of the OTP sent to the patient’s EHR phone number provides a high degree of confidence that the binding is valid. However, we did not see use by patients of the Single-FILE portal after sign-up. We hypothesize that patients typically use the patient portal when they receive an email or text from the site that an appointment is upcoming or laboratory results are available, which then takes them directly to an EHR portal or app on a mobile device and not to Single-FILE. In other words, the use of the patient portal is typically reactive rather than proactive, which limited the use of Single-FILE as we implemented it via a webportal. However, regardless of how the patients access their EHR records (via a webportal or a mobile app), log-in credentials are still required at some point, and we demonstrated that those log-in credentials could be safely associated with a federated identity such as one used for social media.

As health information technology has evolved, the value of access to HPO-specific patient portals [33,34] via a browser is being superseded by access via mobile devices that make it easier for patients to access their EHR data. As previously discussed, there are vendor-specific patient portal solutions that aggregate all of a patient’s EHR data onto an app; thus, this has the same functionality as SSO if the patient stays within that vendor’s ecosystem. In addition, there are some cross-vendor solutions that allow aggregation of EHR data across different vendors, as well as PHR systems that aggregate data from HPOs and other data sources such as pharmacies and fitness trackers. However, these solutions may not readily allow the use of federated credentials for access. The patients we interviewed for this study showed that they appreciated the convenience of using their social credentials to access their EHR data and that remembering EHR portal credentials was a hindrance to access. As previously discussed, biometric authentication may ease the friction associated with access; however, ultimately, log-in credentials are needed, either at the initial configuration for biometric access, when a password reset has been performed, or if an app or mobile device has been upgraded.

When the Single-FILE was being developed, SMART on FHIR was an emerging technology and not widely supported by EHR vendors; therefore, we developed a web-based proof of concept based on the APIs provided by Epic. At the time, we realized that expanding the concept to other EHR vendors would require additional software development as each EHR vendor would have different APIs. SMART on FHIR technology is now stable, and we have successfully replicated the binding of a social identity to an EHR identity by using the patient’s log-in to an EHR portal and FHIR calls to retrieve the patient’s phone number for an OTP challenge or response. The use of SMART on FHIR has the advantages of being vendor agnostic and more robust with respect to EHR software upgrades.

Furthermore, with the adoption of the Interoperability and Patient Access Final Rule (CMS-9115-F) and efforts by the ONC, FHIR has been identified as the basis for secure data exchange via APIs. These standards will foster the development of applications that aggregate health data from a variety of sources in addition to the traditional EHR. If these applications provide support or federated identities, they will enhance the ability of patients to get a holistic, longitudinal view of their EHR data without requiring yet another set of credentials for access.

### Conclusions

In this pilot project, we demonstrated that patients could use an identity they are comfortable with (ie, social identity and associated credentials) as a federated identity to safely ease the friction associated with access to EHR data as they are more likely to access social media more frequently than an EHR or even a PHR portal. Another important feature we built into our pilot software was the ability to use MFA, which provides an additional layer of protection in case one’s log-in credentials are stolen or compromised. Although our solution involved the use of a webportal, the same approach can be used for an app on any mobile device.

This pilot illustrated the need for all participants in a federated identity management system to have high-level organizational support to ensure timely implementation and ensure compatibility with EHR software upgrades. Most of the barriers we encountered can be rendered moot if the support for a federated identity is incorporated into the EHR software and if the EHR vendors adhere to open standards. This is being driven by the ONC’s effort to have EHR vendors incorporate support for FHIR in their software, and it has the added advantage of removing vendor-specific dependencies.

## Abbreviations

API: application programming interface
CSMC: Cedar-Sinai Medical Center
EHR: electronic health record
FHIR: fast healthcare interoperability resource
HPO: health provider organization
IdP: identity provider
MFA: multifactor authentication
NIST: National Institutes of Standards and Technology
ONC: Office of the National Coordinator for Health Technology
OTP: one-time passcode
PHR: personal health record
PRAM: privacy risk assessment methodology
Single-FILE: Single Federated Identity Log-in for electronic health records
SMART: Substitutable Medical Applications, Reusable Technologies
SSO: single sign-on
